# Clearing the airway? A pilot cadaveric study of the LifeVac™ device

**DOI:** 10.1016/j.resplu.2025.101099

**Published:** 2025-09-11

**Authors:** Thomas L. Haupt, Lisa Chionis, Kevin H. Wang

**Affiliations:** Department of Otolaryngology–Head and Neck Surgery, Kaiser Permanente Oakland Medical Center, Oakland, CA, USA

**Keywords:** Airway obstruction, Aspiration, Choking, Resuscitation, Foreign body aspiration

## Abstract

**Background:**

Foreign body aspiration (FBA**)** is a leading cause of accidental death, especially among children and older adults. Suction-based airway clearance devices, such as the LifeVac™, an FDA-registered Class II medical device, have garnered attention as potential adjunctive tools in FBA management; however, data on their efficacy remain limited. This study evaluated the performance of the LifeVac™ in a cadaveric model simulating complete upper airway obstruction by commonly aspirated food items.

**Methods:**

Fresh cadavers with intact upper airway anatomy were used. Grapes, hot dog slices, and steak cubes were placed at the level of the true vocal folds under video-laryngoscopic guidance. Interventions were performed by a PGY-1 otolaryngology resident and a board-certified head and neck surgeon, following LifeVac™ manufacturer instructions. The primary outcome was successful foreign body removal within four minutes. Secondary outcomes included time to removal and evidence of oropharyngeal trauma. All trials were video recorded for technique verification and injury assessment.

**Results:**

Across 21 trials performed on three cadavers, only one successful extraction occurred (1/21, 4.8 %), involving a hot dog segment removed by a PGY-1 resident. One cadaver was edentulous, which resulted in a poor mask seal and ineffective suction. No grapes or steak were successfully extracted. No visible oropharyngeal trauma was identified.

**Conclusion:**

In this cadaveric model, LifeVac™ demonstrated limited efficacy, with successful removal occurring in only one instance. It is unclear if the lack of efficacy is due to device design or the limitations of the cadaveric model. Further research in physiologic or clinical settings is warranted.

## Introduction

Foreign body aspiration (FBA) is the fourth leading cause of accidental death in the United States, especially among young children and older adults.^1^ Commonly aspirated items include soft foods such as grapes and hot dogs. Incidence peaks in children under four years of age and again in older adults.^2^ FBA can result in serious complications, including hypoxia, cardiac arrest, and death.

Current FBA management follows a stepwise approach, beginning with spontaneous coughing and escalating to back blows, abdominal thrusts, and chest compressions if needed.^3^ While this sequence is endorsed by the International Liaison Committee on Resuscitation, the supporting evidence is of very low certainty.^4^

In recent years, suction-based airway clearance devices such as LifeVac™, an FDA-registered Class II medical device, have gained popularity as adjunctive tools for choking emergencies. These portable, non-powered devices are marketed for use in public spaces such as schools, restaurants, and airports. Despite increasing commercial adoption, including reports on Amazon of over 10,000 units sold in the past month and more than 20,000 consumer reviews,^5^ independent data on their safety and effectiveness remains limited. Anecdotal endorsements often include sentiments such as “could really save a life” or “better safe than sorry,” but lack clinical validation.

A systematic review by Dunne et al. reported a 94.3 % first-attempt success rate with the LifeVac™ device but rated the overall quality of evidence as very low due to methodological limitations and probable reporting bias.^6^ Similarly, McKinley et al. described success in 38 of 39 self-reported choking cases involving patients with oropharyngeal dysphagia, though the data were retrospective and lacked external validation.^7^ Conversely, a cadaveric study by Ramaswamy et al. reported very different results: the LifeVac™ was able to remove only soft materials such as moist saltine crackers but failed to extract firmer items like grapes and cashews.^8^ The manufacturer responded by attributing these negative findings to user error and methodological flaws.^9^

Despite varying testimonials and inconsistent results from previous studies, the present study aimed to address these concerns by evaluating the performance of the LifeVac™ in a controlled, fresh (<2 weeks postmortem), non-embalmed cadaveric model simulating complete upper airway obstruction. We adhered strictly to the manufacturer’s recommended push–pull protocol and included anatomical variability across specimens to assess the device under realistic conditions.

## Methods

This study was granted exemption from institutional review by the local ethics board. Data collection was conducted over a single day. Three fresh adult cadavers with intact upper airway anatomy, preserved within two weeks postmortem, were selected. All cadavers were non-embalmed and stored under standard refrigerated conditions until the time of study. Subjects included two females (aged 79 and 80 years) and one male (aged 81 years).

Cadaver access was limited due to institutional availability, which restricted the sample size and procedural repetition. As a pilot investigation, this study was designed to assess the feasibility and performance of a suction-based airway clearance device in a controlled cadaveric model, rather than to establish definitive efficacy. Efforts were made to incorporate variation in body habitus and sex when possible.

Three commonly aspirated food items were tested: whole grapes, cylindrical hot dog segments, and diced steak boluses. Each was positioned firmly into the larynx between the true vocal folds using a cup or alligator forceps under direct visualization with a Glidescope® video laryngoscope.

Two operators participated in the study: a PGY-1 otolaryngology resident and a board-certified head and neck surgeon. Each operator performed an equal number of LifeVac™ trials across all food items and cadavers, following manufacturer instructions. The standardized “push–pull” technique involved fully depressing the plunger to create a vacuum seal, then rapidly pulling upward to generate negative pressure. Each trial involved up to five push–pull cycles followed by video-laryngoscopic reassessment. Additional attempts were permitted until a four-minute threshold was reached. A four-minute trial duration was chosen as it approximates the 3–5-min window in which ischemic depolarization and early irreversible neuronal injury occur following anoxic brain injury, as described in preclinical and guideline-based data.^10^ Timing was standardized using a stopwatch, and the time to successful displacement was recorded when applicable. Devices were reused between trials, as sterility was not required for cadaveric procedures.

A total of 21 trials were conducted. Eighteen trials were performed with an unoccluded esophagus across all three cadavers, with each food item tested by both operators per cadaver (Table 1). Due to concerns that a patulous esophagus may have limited negative-pressure generation, a limited pilot was conducted on Cadaver 1 with the esophagus occluded using a large steak bolus to simulate a sealed distal airway. Three such trials were performed (Table 2).

**Table 1:** Un-occluded esophagus trials.

Cadaver ID	Sex/Age	Operator(s)	Food Items Tested	# Trials	Successful Extractions
Cadaver 1	Female/79	PGY-1 & Attending	Grape, Hot Dog, Steak (×2 each)	6	1 (Hot Dog by PGY-1)
Cadaver 2	Female/80	PGY-1 & Attending	Grape, Hot Dog, Steak (×2 each)	6	0
Cadaver 3	Male/81	PGY-1 & Attending	Grape, Hot Dog, Steak (×2 each)	6	0
Total				18	1 (5.6 %)

**Table 2:** Occluded esophagus trials.

Cadaver ID	Sex/Age	Operator(s)	Food Items Tested	# Trials	Successful Extractions
Cadaver 1	Female/79	Attending	Grape, Steak	3	0
Total				3	0 (0 %)

The primary outcome was dislodgement of the foreign body from the larynx into the hypopharynx within four minutes. The secondary outcome was orofacial trauma attributed to device use, including lacerations, abrasions, or edema from mask pressure. All trials were video recorded using an iPhone 14 to confirm adherence to protocol.

## Results

Of the 18 trials performed with an unoccluded esophagus (Table 1), only one resulted in successful foreign body extraction. A hot dog segment was dislodged from Cadaver 1 by the PGY-1 resident after approximately two minutes of LifeVac™ use. No grapes or steak boluses were successfully removed in any of the unoccluded trials. Cadaver 3, an 81-year-old edentulous male, was unable to form an adequate mask seal, resulting in complete device failure across all trials involving this subject.

Three additional trials were performed on Cadaver 1 with the esophagus occluded (Table 2). Operators subjectively noted increased resistance on the device handle, suggestive of greater negative pressure; however, all occluded trials failed to dislodge the foreign body.

In total, 1 of 21 trials (4.8 %) resulted in successful foreign body extraction. No visible oropharyngeal or facial trauma was identified on post-trial video laryngoscopy or gross inspection.

## Discussion

This pilot cadaver study evaluated the LifeVac™ suction device's ability to dislodge commonly aspirated food items. Strengths of the study include the use of anatomically preserved cadavers (<2 weeks postmortem), biologically relevant food boluses (grapes, hot dog, steak), and strict adherence to manufacturer technique.

Despite standardized technique and controlled conditions, only one of twenty-one trials resulted in successful foreign body removal. Neither grapes nor steak were successfully extracted in any scenario, including under simulated physiologic resistance when esophageal occlusion was applied to mimic muscular tone. These findings suggest substantial limitations in the effectiveness of suction-based devices when applied to firm, slippery, or irregularly shaped food items.

Unlike previous studies that have been critiqued for inconsistent technique,^8^ our methods followed manufacturer protocols precisely. All trials were video recorded and independently reviewed by members of the study team to confirm adherence. Even with correct use, the low success rate raises concerns about the generalizability of the device's performance in real-world clinical settings.

Several studies have reported higher success rates with LifeVac™, including among lay users.^11^, ^12^, ^13^ For example, a randomized crossover manikin trial by Patterson et al. found that LifeVac™ outperformed abdominal thrusts, while the DeChoker™ did not.^14^ However, manikins do not accurately replicate human tissue compliance, mucosal friction, airway secretions, or anatomic variability. These differences may contribute to the overestimation of device efficacy in simulation studies.

Real-world case series, such as that by Dunne et al., have also reported high success rates.^6^ However, these reports are subject to self-report bias, lack of independent verification, and unclear denominators. In contrast, cadaveric studies, while imperfect, offer a more objective platform for evaluating device performance.

Several cadaver-specific factors likely contributed to the limited success in this study. Cadavers do not generate dynamic airway pressures or have airway secretions that aid in food dislodgement. The absence of esophageal tone limits negative pressure generation and does not provide the posterior seal needed to oppose negative pressure. To address this, we piloted esophageal occlusion in a single cadaver, which subjectively increased resistance and perceived suction force but failed to improve extraction success.

A clinically significant finding was the failure to achieve a mask seal in a fully edentulous cadaver. Edentulism is associated with impaired swallowing and an increased risk of aspiration. This is particularly relevant for older adults, who represent a high-risk population for FBA.^15^ In these individuals, the inability to achieve an adequate seal may render the device ineffective.

Only two prior cadaveric studies on LifeVac™ have been published: Juliano et al.^13^ and Ramaswamy et al.^8^ Juliano et al. reported 49 of 50 successful removals of tethered clay spheres placed into a cadaver airway without laryngoscopy. However, these boluses were unrealistic, were not seated in the larynx, and were attached to strings. Movement alone could have dislodged them. The success criteria were not clearly defined, and images from the study showed large neck incisions, raising concerns about potential disruptions to airway anatomy.

Ramaswamy et al. used videofluoroscopy and found that both LifeVac™ and DeChoker™ could move barium-moistened crackers but not grapes or cashews^8^. Whether moistened crackers represent realistic obstructions is unclear. A rebuttal by LifeVac Australia claimed device misuse and inadequate esophageal tone as confounders.^9^ In response, our study followed the company-recommended technique and included esophageal occlusion to simulate tone.

## Limitations

This study was designed as a pilot to assess the feasibility of using fresh, non-embalmed cadavers for evaluating suction-based airway clearance devices. While it offers useful preliminary insights, several limitations affect its generalizability.

The small number of cadavers and trials limits statistical power and broader applicability. All specimens were elderly adults, which reduces relevance to pediatric populations, a group at high risk for choking. Additionally, cadaveric models cannot replicate key physiologic processes such as airway recoil, secretions, and reflexive coughing, which may influence device effectiveness in living subjects.

The absence of a comparator intervention, such as abdominal thrusts or back blows, limits interpretation of relative efficacy. Although all trials were video recorded to verify the technique, the recordings were reviewed by members of the research team rather than independent external reviewers, which introduced potential bias.

Standardization of food impaction was also challenging. Food items were placed under direct visualization, but some fragmented or settled loosely. This raises the possibility that dislodgement in other studies may have occurred due to incidental movement rather than true suction. Suction force was not quantified, and the range of food consistencies tested was limited to three items.

One cadaver was fully edentulous, preventing an effective mask seal and resulting in complete trial failure. This highlights a possible limitation in patients with similar anatomic features. Finally, the 4-minute trial duration may exceed realistic bystander response times, and shorter cutoffs (e.g., 1–2 min) may better reflect clinically relevant outcomes. Overall, while cadaveric models provide valuable anatomic realism, they may not fully simulate clinical scenarios. This pilot study lays the groundwork for future research and highlights the need for larger, controlled studies in high-fidelity simulation or clinical settings.

## Conclusion

In this cadaver-based study, the LifeVac™ device successfully extracted a foreign body in only one of twenty-one trials. The role of suction-based devices in first-aid algorithms for choking remains uncertain. Further research using high-fidelity simulation models or prospective clinical studies is warranted to better define their utility in airway emergencies.

## Credit authorship contribution statement

**Thomas L. Haupt:** Writing – review & editing, Writing – original draft, Visualization, Validation, Methodology, Investigation, Formal analysis, Data curation, Conceptualization. **Lisa Chionis:** Writing – review & editing, Project administration, Methodology, Data curation, Conceptualization. **Kevin H. Wang:** Writing – review & editing, Writing – original draft, Visualization, Validation, Supervision, Formal analysis, Data curation, Conceptualization.

## Funding

None.

## Declaration of competing interest

The authors declare that they have no known competing financial interests or personal relationships that could have appeared to influence the work reported in this paper.
